# Freeze‐dried, oven‐dried, and microencapsulation of essential oil from *Allium sativum* as potential preservative agents of minced meat

**DOI:** 10.1002/fsn3.1487

**Published:** 2020-02-27

**Authors:** Hanen Najjaa, Raja Chekki, Walid Elfalleh, Hajer Tlili, Sana Jaballah, Nabiha Bouzouita

**Affiliations:** ^1^ Laboratory of Pastoral Ecosystems and Valorization of Spontaneous Plants and Microorganisms Institute of Arid Regions (IRA) Medenine Tunisia; ^2^ Ecole Supérieure des Industries Alimentaires Tunis Tunisia; ^3^ Département de Transfert Technologique Centre Technique de la Chimie Tunis Tunisia; ^4^ Laboratoire Energie Eau Environnement et Procèdes Ecole Nationale d'Ingénieurs de Gabès Université de Gabès Gabès Tunisia; ^5^ Laboratoire d'analyses et d'essais Centre Technique des industries Agro‐alimentaire Tunis Tunisia

**Keywords:** *Allium sativum*, antimicrobial, freeze‐drying, microcapsules, oven‐drying

## Abstract

The present study was conducted to compare the antibacterial activity of oven‐dried and freeze‐dried *Allium sativum* along with its spray‐dried microencapsulated essential oil in the preservation of minced beef meat. *Allium sativum* extracts were tested against mesophilic aerobic microorganisms, coagulase‐positive *staphylococci*, *Escherichia coli*, *Salmonella* sp., and the sulfite‐reducing anaerobes. A difference between the chemical compositions of powders obtained by the conventional oven‐drying and freeze‐drying has been verified by HPLC‐MS^2^, freeze‐dried fresh garlic powder contains 74% of allicin, and 12% cysteine sulfoxides comparing to the oven‐drying garlic powder in which is detected two thiosulfinate isomers: allicin (67%) and allyl‐1‐propenyl thiosulfinate (21%). CIELAB color analysis was performed to assess the effect of drying temperature on powders. The microflora‐inhibiting effect of freeze‐dried fresh garlic and the spray‐dried microencapsulated essential oil at a concentration of 20% represents a promising way to be used in food systems such as meat and meat products preservation, at 4–8°C.

## Graphical abstract

The microflora‐inhibiting effect of freeze‐dried fresh garlic and the spray‐dried microencapsulated essential oil at a concentration of 20% represents a promising way to be used in meat preservation, at 4–8°C.

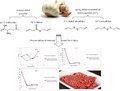

## 1 INTRODUCTION

Meat and its derivatives are foods which can be easily spoiled, frequently associated with toxinfection (Roberts & Weese, 1998). The contamination of meat begins at the slaughter‐house and continues during the operations of deboning and meat preparation in butcheries (Oumokhtar, Berrada, Ameur, & Fakir, 2008; Roberts & Weese, 1998)*.* The initial mesophile microflora of the meat ranges between “10^2^” and “10^3^” UFC cm^‐2^ (Borch, Kant‐Muermans, & Blixt, 1996). In general, the more meat is cut into smaller pieces, the more its shelf life decreases (Njue, Okemo, & Monda, 2009; Torreggiani, Lucas, Blond, & Raoult‐Wack, 1999)*.*Contamination can be related to poor hygienic quality products, serious food toxinfections, and serious economic losses (Ghafir & Daube, 2007; Roberts & Weese, 1998)*.* The pathogenic bacteria, such as *Salmonella typhi (S. typhi)*, *Escherichia,* and *Staphylococcus aureus* (*S. aureus*), are the main etiologic agents of diseases caused by contaminated meat (Bailly, Brugère, & Chardon, 2012)*.* Hence, deterioration in quality and potential public health issues are common if these products are not properly handled and preserved (Fratianni et al., 2010). Therefore, consumers prefer natural food additives over traditional synthetic preservatives (Hyldgaard, Mygind, & Meyer, 2012; Mariutti, Nogueira, & Bragagnolo, 2011), thereby driving research for identifying natural food additives with broad‐spectrum antimicrobial activities. These additives may also improve the quality and shelf life of meat products.

Garlic has a wide spectrum of actions, not only antibacterial, antifungal, and antioxidant, but also it has beneficial effects on the cardiovascular and immune systems (Harris, S., S., & D., 2001). During the last decade, the antimicrobial activity of garlic, garlic‐derived organosulfur compounds, and essential oil was widely investigated against both food spoilage bacteria and foodborne pathogens (Ankri & Mirelman, 1999; Benkeblia, 2004; Bronwyn, Hughes, & Lawson, 1991; Leuschner & Ielsch, 2003). However, the organosulfur compound volatility and low physic‐chemical stability limit the possibility of its application as a food‐functional ingredient. Dried garlic powder contains important amount of alliin (sulfoxyde S‐allylcysteine) (approximately 1%). The highest rate of alliin to allicin conversion has been observed in the temperature range of 35–36°C (Lanzotti, 2006; Mishra, Upadhyay, & Maheshwari, 2001)*.* The retention of allicine has been found to be more specific in freeze‐dried samples at a temperature of 20°C (Ratti, Araya‐Farias, Mendez‐Lagunas, & Makhlouf, 2007). According to two studies of garlic preparations, allicine decreased to nondetectable amounts within 1–6 days (Yu & Wu, 1989). This observation has directed our attention to the importance of choosing the adequate technique of drying. Garlic and its extracts preservation was carried out through several drying techniques, that is, hot air‐drying (in fixed and fluidized bed) and freezing‐drying (Aware & Thorat, 2011; Bhandari & Howes, 1999). Foodstuff preservation by drying methods should be aimed at the preservation of both its mechanical properties and chemical composition in such a manner that after the rehydration of the dried product, it will maintain its gustatory value (Acosta‐Esquijarosa, Álvarez‐Reyes, & González‐Lavaut, 2011; Kramkowski, Kamiński, & Serowik, 2001)*.*

Freeze‐drying is a process which can preserve the quality of fresh *A. sativum* by minimizing the chemical reactions, biological and microbiological deterioration (Singh, Singh, & Singh, 2014), and its degradation under high temperature. A study conducted by Puranik, Srivastava, Mishra, and Saxena (2012), on Indian garlic, showed that the freeze‐dried sample had a maximum loss in moisture of 58.8%, with a higher quality score, followed by fluidized‐bed drying, oven‐drying, and heating by microwaves. Sablaniet al. (2007) demonstrated that porosity is affected significantly by drying techniques. It has been also authorized that the sample size and temperature significantly influenced the freeze‐drying duration and the remaining moisture content of garlic samples (Aware & Thorat, 2011)*.* Tests proved that more than 95% of the antioxidant activities remained in the freeze‐dried preparations (Leelarungrayub, Rattanapanone, Chanarat, & Gebicki, 2006) and the preservation of the allicine is more effective in freeze‐dried samples at 20°C (Ratti et al., 2007)*.* According to the same study, garlic maintains its intact capacity but the retention of allicine decreases when the whole bulbs are dried. However, the decrease is not significant in sliced garlic samples (Chen & Mujumdar, 2009).

The major studies have interested to the antimicrobial activity of fresh garlic and its extracts, and few studies have focused to antimicrobial activity of garlic essential oil (EO), spray‐dried microcapsules, oven‐dried, and freeze‐dried forms. The antimicrobial activity, of garlic freeze‐dried and essential oils, against various disease‐causing agents like *Bacillus subtilis, Pseudomonas aeruginosa*, *S. aureus (S. aureus)*, *E. coli*(*E. Coli*), and *Proteus spp*. was investigated (Viswanathan, Phadatare, & Mukne, 2014). Al‐Delaimy and Ali (1970) showed that freeze‐dried garlic powder has an inhibiting effect against *B. subtilis*, *S. aureus,* and *Streptococcus mutans*. This powder is characterized by the presence of thiosulfinates other than the allicin. Yin and Cheng (2003) reported a significant decrease of the total aerobes and prevented growing of the pathogenic bacteria as *S. typhimurium*, *E. coli*, *Listeria monocytogenes*, *S. aureus,* and *Campylobacter jejuni* in minced beef treated by four garlic thiosulfinates. A reduction in the psychrotrophic bacteria was noticed while using the garlic predried in irradiated and nonirradiated steaks (Benkeblia & Lanzotti, 2007)*.* In addition, it was observed that the initial aerobic flora of dry chicken sausage was significantly reduced by fresh garlic and garlic powder treatment, and its shelf life was considerably extended to 21 days. The potential use of fresh garlic and garlic powder in food preservation was mentioned (Sallam, Ishioroshi, & Samejima, 2004). Minced meat is a product which is quickly contaminated at room temperature. Its lifetime is estimated as two days when preserved in a conventional refrigerator and under aerobic atmosphere (Millette, 2003).

In the current study, we tried to extend the lifetime to 6 days. Using various microorganisms of hygienic interest, we investigated the effect of freeze‐dried garlic powder, oven‐dried powder, and garlic essential oil (EO) microcapsules on the shelf life of minced beef meat marketed in Tunisia.

## 2 MATERIALS AND METHODS

### 2.1 Sample collection and preparation

The garlic (*A. sativum*), belong to “Softneck” variety, used in this study was identified by Dr. Nadia Ghrab and harvested from the agricultural zone of the Manouba region, during the month of October 2015. The samples were stored until used in the experiments of the drying processes. The final moisture content of the fresh *A. sativum* was 64 ± 0.2% using a Mettler Toledo HB43 halogen dryer at 105°C to a constant mass.

### 2.2 Essential oil extraction

The essential oil (EO) of *A. sativum* was obtained by steam distillation. Thirty kg of *A. sativum* was distilled for 4 hr at 80°C and the oil obtained was dried with anhydrous sodium sulfate and stored in full, dark vials at + 4°C to be used in the encapsulation. Essential oil yield of *A. sativum* established on a wet weight basis was 0.27%.

### 2.3 Convective air‐drying

Fresh garlic samples were cut into small pieces by knife and dried in an oven with ventilation at 50°C for 5 hr. The final moisture content of the powder obtained Oven‐drying by convection was 6.7 ± 0.5%.

### 2.4 Freeze‐drying

Garlic bulbs were stored at room temperature (20°C) until their use. Before placing the samples into freeze‐drying room, the samples were cut into small pieces by knife and frozen at −80°C for 24 hr. The frozen samples were then transferred to a freeze dryer (CHRIST Alpha 1–4 LSC). A pressure of 0.94 mbar and a temperature of‐5°C were applied for 72 hr (Sablani et al., 2007). The final moisture content of the powder obtained freeze‐drying was 6.3 ± 0.1%.

### 2.5 Encapsulation of essential oils

Preparation of the microcapsules in the emulsion was carried out by oil in water (O/W). Aqueous solutions were prepared using maltodextrin (MDX) (70% W/V) and gum Arabic (AG) (30% W/V) as carrier (wall material). MDX and AG were previously dissolved in distilled water at 50 C stirred with heater–stirrer and left to stand for 12 hr at room temperature. For the emulsion coacervate preparations, four different concentrations of garlic EO (5, 10, 15, and 20%) were incorporated into the wall material suspension using an Ultraturrax homogenizer at 24,000 rpm for 30 min. The emulsion was sprayed by a laboratory spray, Buchi 290 “Minisprayer Advanced” using 180°C (5, 10 and 15%) and 200°C (20%) inlet temperature of drying air and 75°C (5, 10, and 15%) and 80°C (20%) outlet temperature. The feed rate of the emulsion 45 ml/min compressed air flow rate is 600 L/h and the drying air flow rate of 90 bars. The emulsions obtained (500 g) were stored at room temperature until use. The encapsulation efficiency, expressed as a percentage of oil entrapped in the microcapsules, was determined according to Devi, Hazarika, Deka, and Kakati (2012). The final microencapsulation efficiency of emulsion used was more than 70%.

### 2.6 Characterization of powders by LC‐MS/MS

One gram of powder (oven‐dried and freeze‐dried garlic) was dissolved in 10 ml of demineralized water, and a characterization with HPLC‐MS/MS Agilent Triples Quad was carried out according to the conditions reported by Arnault et al. (2003). Ions [M + H] ^+^were formed by using ESI in positive mode. The operation parameters of the mass spectrometer were the following: temperature of 250°C, tension of the atomizer of 5.0 kV, and energy of collision of 23 eV. Acquisition was carried out in mode MRM according to the (Table S1).

For allicin analysis, its thiosulfinate isomers which coexist were considered (Table S2) (Ilić et al., 2012; Sablani et al., 2007)*.* Other by‐products were envisaged like vinyldithiins and ajoene which were compounds of degradation of the allicin. Their distinction was based on their m/z fragment abundance (Table S3).

### 2.7 Characterization of microcapsules by GC‐MS

The essential oil microcapsules were treated with dichloromethane (25 ml) and stirred for 10 hr. After extraction of the essential oil of *A. sativum*, the solvent was evaporated in an oven, at 40°C. The qualitative analysis of essential oils was carried out by gas chromatography coupled to mass spectrometry (GC‐MS: Agilent 6890N) using HP‐5 column (5% phenyl methyl siloxane). Dimensions: 30 m × 250 µm × 0.32 μm. Temperature program: 40°C/5 min, 40–120°C at 5°C/min and from 120 to 200°C at a rate of 8°C/min. The carrier gas was helium with a flow rate of 1 (ml/min). The source of the mass spectrometer had a temperature of 300°C and mass ranged from 50 to 350 amu. The identification of the compounds present in the essential oil was carried out by the Wiley 275 L spectral library.

### 2.8 Determination of the CIELAB chromatic characteristics of the powders

Chromatic characteristics of the products were determined by colorimetry corresponding to clearness (L*), component of red/green (a*), and component of yellow/blue color (b*). The parameters of color were given by using a Gardner 45/0 (Germany) type colorimeter. The samples were versed in a clear glass, and the color parameters (L*, a*, and b*) were recorded.

### 2.9 Enumeration of microorganisms

Sample preparation was performed according to ISO 6887–1:2003, in aseptic and sterile conditions. A total of 25 g of ground meat was homogenized. The initial suspension was prepared by using a sterile spatula and by suspending a test portion of 10 g of ground material in 90 ml of buffered peptone water (BPW) and homogenized for 2 min. Various dilutions were prepared: 10^–1^, 10^–2^, 10^–3^, and 10^–4^ in test tubes filled with 9 ml of tryptone salt (TS). The investigated powders and microcapsules were dissolved in DMSO/H_2_O = 50:50 (1 mgml^‐1^) and were administered in the form of inoculum. The final concentration of freeze‐dried, oven‐dried, and spray‐dried extracts after inoculation was 0.1%. After incubation, the counting was done according to the following standards: total mesophilic aerobic microorganisms GAMT obtained at 30°C (ISO 4833‐1/2013), the coagulase‐positive *staphylococci* at 37°C (ISO 6888–1–1999), sulfite‐reducing anaerobes (CSR) (ISO 15,213:2003), *Salmonellas *(ISO 6,579:2002), and *E. coli* (*E. coli*) (ISO 16649–2). The most reliable results were obtained from the plates containing from 10 to 300 colonies (*ISO7218:2007‐*Microbiology of the food stuffs and feeding stuffs‐General rules relating to the microbiological analyses). The obtained results were interpreted according to the following classes:

- x ≤ M: Satisfactory food;
- m ≤ x ≤ M: Acceptable food
- x ≥ M: Unacceptable food where x = obtained concentration log CFUg^‐1^
- m = limit below which all results are considered satisfactory and *M* = acceptability limit beyond which the results are no longer considered satisfactory.

### 2.10 Statistical analysis

All analytical determinations were performed in triplicate for three samples (*n* = 3). One‐way analysis of variance (ANOVA) was conducted using SPSS software, and 17.0. Duncan's multiple range test (*p* < .05) was used to compare the average responses between treatments.

## 3 RESULTS AND DISCUSSION

### 3.1 Comparison of the drying processes of garlic

Oven‐drying by convection and freeze‐drying were studied as potential processes to preserve and concentrate the allicine in garlic. Allicin constitutes a principal compound of the thiosulfinates (*M* = 162 gmol^‐1^) in garlic and is the main antimicrobial compound of fresh crushed garlic (Ratti et al., 2007). Allicin is very unstable and its decomposition proceeds through several pathways and the differentiation among its isomers are difficult. The two major known isomer fragments were identified by MS/MS, based on the abundance of various selected fragments m/z: 121 and 73. Table 1 shows that the tested freeze‐dried fresh garlic powder contains 74% of allicin. Cysteine sulfoxides (12%) and vinyldithiins (7%) were also detected. The absence of the glutamylcysteines could is explained by the decomposition of γ‐glutamylcysteine, under temperatures lower than 0°C (Benkeblia & Lanzotti, 2007)*.* The chemical analysis of oven‐dried garlic powder (Table 2) recorded two thiosulfinate isomers: allicin (67%) and allyl‐1‐propenylthiosulfinate (21%). The presence of the vinyldithiines (5%) and the ajoene (3%) were also detected. The composition of oven‐dried garlic and freeze‐dried garlic samples was summarized in (Table 2).The allicin content, in the oven‐dried powder, decreased compared to the freeze‐dried garlic samples. This agrees with that of Ratti et al.(2007), who found that allicin content decreases with an increase of drying temperature. According to Shi, Maguer, and Bryan (2002), temperature contributes to both the formation of thiosulfinates generated by alliinase and its decomposition proceeds through several pathways.

**Table 1:** Identification of the allicin in freeze‐dried (FD) oven‐dried (OD) garlic powder

Peak	Retention time (min)	Denomination	FD Relative %	OD Relative %
1	1.5	Isoalliin	5.5	—
2	5.1	Alliin	3.8	—
3	6.4–6.5	2‐vinyl‐[4H]−1.3‐dithiin	3.8	3.1
4	9.3–9.9	3‐vinyl‐[4H]−1.2‐dithiin	3.0	2.0
5	10.3	Methiin	3.1	‐‐
6	12.5–13.9	*N*.I	3.3	1.8
7	15.9	SAC (S‐Allyl‐L‐cysteine)	3.8	2.3
8	19.3	Ajoene	—	3.0
9	20.3	Allyl−1‐propenyl thiosulfinate (E.Z)	—	20.7
10	29.6	Diallyl thiosulfinate (Allicin)	73.7	67.1

Abbreviation: N.I, Not identified.

**Table 2:** Comparison of freeze‐dried and oven‐dried garlic composition

	Freeze‐dried garlic (relative %)	Oven‐dried garlic (relative %)
*Sulfoxides*	12	0
*SAC*	4	2.3
*Vinyldithiins*	7	5
*Ajoene*	0	3
*Allicin*	74	67
*Allicin isomers*	0	21

Under selective conditions, the vacuolar enzyme alliinase transforms “alliins” into the very unstable thiosulfonates yielding 1‐propenyl contains sulfonates. It was recently shown that at high temperatures, poly‐sulfurous compounds are formed containing up to five sulfur atoms. Thus, the presence of sulfoxides in freeze‐dried garlic (12%) could be explained either by the inactivation of allinase, an enzymatic cleavage that may lead to thiosulfinates according to reaction (1) (Shi et al., 2002).

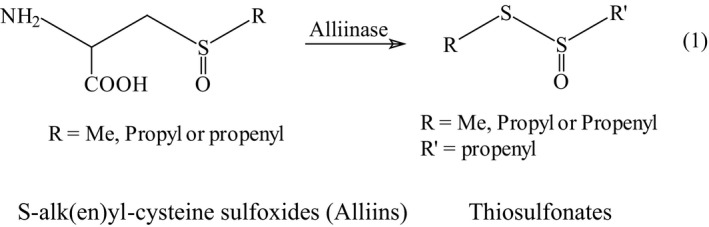

Color is an important characteristic used to compare the food aspects of garlic and it is frequently used for evaluating the products' acceptance (Kang, Yong, Ma'aruf, Osman, & Nazaruddin, 2014)*.* In our experiments, we measured the parameters of clearness (L*), the component of the red/green (a*), and that of the yellow/blue color (b*). The results are presented in (Table 3). The highest clearness value (88.06) was observed in the freeze‐dried garlic powder indicating its high rate of clearness as compared to that of oven‐dried garlic.

**Table 3:** CIELAB color parameters for freeze‐dried garlic and oven‐dried garlic

Color parameters	Freeze‐dried garlic	Oven‐dried garlic
L*	88.06 ± 0.01a	72.36 ± 0.04b
a*	1.54 ± 0.05b	7.88 ± 0.03a
b*	19.23 ± 0.04b	34.23 ± 0.07a

Note

Data presented as the mean ± standard deviation (*n* = 3). Within a row, means lacking a common lowercase letter are different at (*p* < .05).

A maximum value of clearness is allotted to the minimal deterioration of the color; consequently, freeze‐drying can delay oxidation and other chemical reactions (Kang et al., 2014). The highest a* value (7.88) was observed in oven‐dried garlic which indicates an increase in the red color. In oven‐dried garlic, a positive and high value of b* was detected (34.23 against 19.23) as compared to freeze‐dried garlic. This indicates the “yellowness effect” of temperature. Indeed, the oven‐drying involved an oxidative degradation and thus to lead to a change of color (darkening) (Kang et al., 2014)*.* Drying process significantly alters color and chemical composition of garlic powder. Staba, Lash, and Staba (2001) showed that freeze‐drying did not affect the chemical composition of fresh garlic cloves.

### 3.2 Characterization of essential oil microcapsules by GC‐MS

The content of organosulfur compounds in EO significantly decreased from 66% to 40% in the EO microcapsules. In fact, by switching to high temperatures (180°C) for spray‐drying, the essential oil is degraded and/or volatilized. This is shown by the predominance of trisulfides (30%). The presence of diallyl sulfide is about 9%. However, application of EOs is partially limited due to their intense aroma which may cause negative organoleptic effects, as well as, their effectiveness is moderate due to their interaction with food ingredients and structure. Novel technologies such as encapsulation of EOs to improve the microbial stability and the sensory quality of meat and meat products are being used in the meat industry.

Besides, maintaining desirable properties of original food products containing natural preservatives for long‐term is an ongoing problem in food industries. Natural herbs or spices tend to lose their efficiency over short‐term storage and they fail to release their beneficial effects (on microbial or physicochemical properties of food products) evenly over a longtime period. Conversely, microencapsulation is defined as a process in which tiny particles or droplets are surrounded by a coating to form small capsules (Jafari, Ghanbari, Dehnad, & Ganje, 2018) and can protect sensitive materials from moisture, heat, light, or oxidation (Khazaei, Jafaria, Ghorbania, & Hemmati, 2014).

### 3.3 Effect of oven‐dried, freeze‐dried and the EO microcapsules in microbiological meat quality

Oven‐dried and freeze‐dried garlic powders and the EO spray‐dried microcapsules were investigated at different concentrations under modified atmosphere in the course of 06 days. No proliferation has been observed for the coagulase *staphylococcus* and *salmonellas* in minced meat. In order to detect the various inhibition levels of different extracts on total aerobic mesophilic flora (GAMT), *E. coli* and sulfite‐reducing anaerobe (CSR) enumeration was determined. These pathogenic microorganisms can result in foodborne illnesses to consumers if the products are not preserved and handled properly. Additionally, foodborne diseases have emerged as important and growing public health and economic problems in many countries over the last few decades. Foodborne diseases are not limited to a particular age group or country (Tauxe, Doyle, Kuchenmuller, Schlundt, & Stein, 2010).

Compared to the control sample, not treated, which stands for a quality unfit for human consumption, during the two days (Table 4), all samples prolong the preservation period of minced meat with satisfactory quality (x ≤ m). Only 20% EO microcapsules and freeze‐dried garlic can extend the shelf life of minced meat of satisfactory quality (x ≤ m) by 4 days. The 15% EO content of microcapsules may also extend the shelf life of minced meat of acceptable quality (m ≤ x ≤ M) by 4 days. The results showed that the inhibitory effect of microcapsules increases with the increase of the essential oil concentration (5–20%). The reactivity of the lyophilized fresh garlic powder is very effective for the inhibition of GAMT during the first 2 days and it is a suitable alternative for inhibiting the total mesophilic aerobic microorganisms (GAMT) during the 06 days of refrigerated storage, at 4–8°C.

**Table 4:** Total aerobic mesophilic flora (GAMT) enumeration in ground meat

Extract*	Day 0 at 4°C	Day 2 at 4°C	Day 4 at 8°C	Day 6 at 8°C	Limits	
					m	M
	log CFU/g					
Control sample	4.0 ± 0.1aC	6.0 ± 0.1aB	8.5 ± 0.3aA	8.5 ± 0.3aA		
Freeze‐dried garlic powder	4.0 ± 0.2aC	3.3 ± 0.5cC	4.4 ± 0.2dB	5.0 ± 0.1dA		
Oven‐dried garlic powder	4.0 ± 0.2aD	5.4 ± 0.1bC	7.3 ± 0.0bA	7.4 ± 0.32cA		
Microcapsules EO 5%	4.0 ± 0.1aD	6.0 ± 0.2aC	7.3 ± 0.1bB	8.2 ± 0.4aA		
Microcapsules EO 10%	4.0 ± 0.2aD	5.7 ± 0.2bC	7.5 ± 0.1bB	8.0 ± 0.6aA		
Microcapsules EO 15%	4.0 ± 0.1aD	5.5 ± 0.1bC	6.2 ± 0.4cB	7.6 ± 0.18bA	5.7	6.7
Microcapsules EO 20%	4.0 ± 0.1aC	5.7 ± 0.15bB	4.1 ± 0.3dC	6.4 ± 0.4cA		

Note

Within a column, means lacking a common lowercase letter are different at (*p* < .05).

Within a row and within each preservation period, means lacking a common uppercase letter are different (*p* < .05).

* Concentration of each extract after inoculation was 0.1%.

From the obtained results, only samples treated for 6 days by freeze‐dried garlic powder decreasing count of aerobic plate microorganisms and prolong the shelf life of satisfactory quality (x ≤ m) when compared to control samples.

The treated samples with different treatment of garlic showed inhibiting the growth of *E. coli* in minced meat (1.7 ≤ x ≤ 2.7), with satisfactory and acceptable quality, during the 06 days in cold storage (Table 5). From the obtained results, lyophilized garlic powder and essential oil microcapsules of 20% had the lowest counts of *E*. *coli* forms compared to control sample (x < 1). This proved that the presence of these garlic ingredients allowed for a significant reduction in the logarithmic number of *E. coli* during the entire shelf life. To extend the period of refrigerated storage, which is the most common method used for preserving fresh meat and meat products, many synthetic additives have been used over the years (Chen, Pearson, & Gray, 1992). Synthetic additives have been accused for some carcinogenic and toxic properties. This increased the consumer concerns toward healthier meat and meat products and the demand for natural food additives over the years, which led researchers to examine natural alternatives to synthetic food additives (Mariutti et al., 2011). These natural additives should improve meat quality without leaving residues in the product or in the environment (Simitzis et al., 2008).

**Table 5:** *Escherichia coli* enumeration in ground meat

Extract*	Day 0 at 4°C	Day 2 at 4°C	Day 4 at 8°C	Day 6 at 8°C	Limits	
					m	M
	log CFU/g					
Control sample	2.4 ± 0.2aB	2.6 ± 0.4aAB	3.2 ± 0.26aA	4 ± 0.5aA		
Freeze‐dried garlic powder	2.6 ± 0.0aA	<1 ± 0.5dB	<1 ± 0.6bB 0	<1 ± 0.3B		
Oven‐dried garlic powder	2.5 ± 0.1aA	2 ± 0.1bB	< 1.6 ± 0.2bC	< 1.6 ± 0.2bC		
Microcapsules EO 5%	2.4 ± 0.2aA	2.6 ± 0.1aA	< 1.6 ± 0.0bB	<1 ± 0.1cC	1.7	2.7
Microcapsules EO 10%	2.3 ± 0.3aA	< 1.6 ± 0.7cB	< 1.6 ± 0.2bB	<1 ± 0.4bC		
Microcapsules EO 15%	2.4 ± 0.2aA	< 1.6 ± 0.7cB	<1 ± 0.4cC	<1 ± 0.4bC		
Microcapsules EO 20%	2.5 ± 0.1aA	<1 ± 0.5dB	<1 ± 0.4cB	<1 ± 0.1cC		

Note

Within a column, means lacking a common lowercase letter are different (*p* < .05).

Within a row and within each preservation period, means lacking a common uppercase letter are different (*p* < .05).

* Concentration of each extract after inoculation was 0.1%.

Table 6 illustrates that the antimicrobial activity of the EO microcapsules of 15% and 20%, freeze‐dried garlic powder, showed lowering in CSR, GAMT, and *E. coli* values for 6 days compared to oven‐dried garlic powder and the EO microcapsules of 5 and 10%. Such findings may be attributed to the high thiosulfinate compounds effect compared to the polysulfides (Ross, O'Gara, Hill, Sleightholme, & Maslin, 2001), which are known for their functional group S‐(O)‐S which is supposed to react with –SH group of cellular proteins and to generate mixed disulfides (Kim, Huh, Kyung, & Kyung, 2004).

**Table 6:** Sulfite‐reducing anaerobes (CSR) enumeration in ground meat

Extract*	Day 0 at 4°C	Day 2 at 4°C	Day 4 at 8°C	Day 6 at 8°C	Limits	
					m	M
	log CFU/g					
Control sample	2 ± 0.1aA	2 ± 0.1aA	2.7 ± 0.1aA	3.5 ± 0.08aA		
Freeze‐dried garlic powder	2.1 ± 0.2aA	<1 ± 0.6dB	<1 ± 0.6cB	<1 ± 0.4bC		
Oven‐dried garlic powder	2 ± 0.1aA	<1.6 ± 0.1bB	<1 ± 0.3C	<1 ± 0.3bC		
Microcapsules EO 5%	2 ± 0.1aA	<1.6 ± 0.45bB	<1.6 ± 0.2bB	<1 ± 0.4bC		
Microcapsules EO 10%	2 ± 0.1aA	<1.6 ± 0.1bB	<1 ± 0.5cC	<1 ± 0.4bC	1.7	2.7
Microcapsules EO 15%	2 ± 0.2aA	<1 ± 0.2B	<1 ± 0.2cB	<1 ± 0.1dC		
Microcapsules EO 20%	2 ± 0.1aA	<1 ± 0.3B	<1 ± 0.2cB	<1 ± 0.1dD		

Note

Within a column, means lacking a common lowercase letter are different (*p* < .05).

Within a row and within each preservation period, means lacking a common uppercase letter are different (*p* < .05).

* Concentration of each extract after inoculation was 0.1%.

A number of reports have indicated that the antimicrobial activity of a given EO can be attributed to its major constituents as well as their interaction with minor constituents present in oils (Hyldgaard et al., 2012). Burt (2004) reported that the antimicrobial activity of EOs is not attributable to one specific mechanism. Sallam et al. (2004) mentioned that antimicrobial benefits of garlic oil is due to her is riches in organosulfur compounds and their precursors (allicin, diallyl sulfide & diallyl trisulfides). Pranoto, Salokhe, and Rakshit (2005) added inhibit the growth of a lot of pathogens as APC, *E. coli* and *S. aureus* by reacting with their cystine, inactivating the thio‐containing enzymes or affecting the metabolism of lipids (Weiguo et al., 2004), and subsequently, extending the shelf life of the product, so the garlic extracts are potentially useful in preserving meat products (Pranoto et al., 2005).

Many studies have examined the use of aromatic phytochemical preparations with dual functionality against microbial spoilage and lipid oxidation in meat and meat products (Bozin, Mimica‐Dukic, Samojlik, & Jovin, 2007), particularly EOs (Govaris, Solomakos, Pexara, & Chatzopoulou, 2010). These nonphytotoxic oils (Al‐Reza, Rahman, Lee, & Kang, 2010) are safe as food additives and certified as “Generally Recognized as Safe” (GRAS) (Lucera, Costa, Conte, & Nobile, 2012), which results in higher consumer acceptability. Numerous experimental applications of EOs have shown their suitability as preservatives in meat and meat products (Bozin et al., 2007), particularly as effective natural antimicrobial agents against foodborne pathogenic and spoilage bacteria (Bajpai, Baek, & Kang, 2012). This study solved some limitations have been identified in the application of EOs in meat and meat products, particularly through hurdle technology using the encapsulation of EOs. The interaction of some EOs with food ingredients and structure may decrease their effectiveness (Skandamis & Nychas, 2001).

This might explain the free‐fall reduction, observed in the first 2 days, for the freeze‐dried garlic powder as compared to the effect of essential oil spray‐dried microcapsules. Also, these results were like those obtained by Rahman et al. (2006), who mentioned that the highest inhibiting effect against all the tested pathogenic strains was fresh garlic followed by the freeze‐dried and oven‐dried garlic powder, at 60°C, respectively. The antimicrobial effect of the various studied treatment depends on the concentration of extracts, storage period, and the microorganism species (Kumar & Berwal, 1998)*.*

## 4 CONCLUSION

Meat and meat products are highly subject to microbial deterioration, which ultimately leads to safety and quality issues if the meat is not properly handled and preserved. Garlic derived as freeze‐dried fresh garlic and the spray‐dried microencapsulated essential oil at a concentration of 20% can be effectively used in meat and meat products as natural alternatives to synthetic food additives, particularly as effective antimicrobial agents. Producers and manufacturers have been challenged by the increasing demand for safe and high‐quality meat and meat products over the past few decades. Particularly, the recent demand for minimally processed, easily prepared, and ready‐to‐eat meat products combined with the novel concepts of all‐natural and clean‐label has rapidly increased. These products may contain natural or organic ingredients without artificial preservatives that do not trigger common food allergies or sensitivities.

## CONFLICT OF INTEREST

The authors declare that they have no conflict of interest.

## ETHICAL STATEMENT

This study does not involve any human or animal testing.

## INFORMED CONSENT

Written informed consent was obtained from all study participants.

## Supporting information

TableS1‐S3
