# Supplementary material for: Freeze‐dried, oven‐dried, and microencapsulation of essential oil from Allium sativum as potential preservative agents of minced meat
Source: Food Sci Nutr. 2020 Feb 27;8(4):1995–2003. doi: 10.1002/fsn3.1487 (PMC7174234; doi:10.1002/fsn3.1487)
Supplement: Supplementary file 1 — TableS1‐S3 [file FSN3-8-1995-s001.docx]

SUPPLEMENTAL MATERIAL

**S1 Table. Selected fragments for HPLC-MS^2^ analysis of *A. sativum***

| Substance | FragmentMS^1^ (M+H)/  (m/z) | FragmentMS^2^/  (m/z) |
| --- | --- | --- |
| *Alliin*  *S-Allyl-L-cysteine sulfoxyde* | 178 | 86 (Sreedhara, 2012) |
| *Methiin*  *S-methyl-L-cysteine sulfoxyde* | 152 | 88 (Ziegert, 2006) |
| *Isoalliin*  *Trans S-1-propenyl-L-cysteine sulfoxyde* | 178 | 88(Ziegert, 2006) |
| *Propiin*  *S-propyl-L-cysteine sulfoxyde* | 180 | 88(Ziegert, 2006) |
| *GSMC*  *γ-glutamyl-S-methyl-L-cysteine* | 281 | 134 (Zhang, 2013) |
| *GSAC*  *γ-glutamyl-S-Allyl-L-cysteine* | 291 | 145 (Arnault, 2003) |
| *GSPC*  *γ-glutamyl-S-(trans-1-propenyl)-L-cysteine* | 291 | 170(Arnault, 2003) |
| *SAC or deoxyalliin*  *S-Allyl-L-cysteine* | 162 | 90 (Nikolić, 2012) |
| *Allicin*  *Diallyl thiosulfinate* | 163 | 73 (Khar, 2011) |

**S2 Table.** **Selected fragments and their abundances for HPLC-MS^2^ analysis of the thiosulfinates of *A. sativum***

| Thiosulfinates | Fragment MS^1^/m/z (abundance) | Fragment 1MS^2^/  m/z (abundance) | Fragment 2 MS^2^/  m/z (abundance) |
| --- | --- | --- | --- |
| Diallyl thiosulfinate (Allicin) | 163(7) | 121 (12) | 73 (100) |
| Allyl-1-propenyl thiosulfinate(E, Z) | 163 (10) | 121 (65) | 73 (10) |

**S3 Table.** **Selected fragments and their abundances for HPLC-MS^2^ analysis of the vinyldithiins of *A.sativum* *(34)***

| Thiosulfinates | Fragment MS^1^/  m/z (abundance) | Fragment1 MS^2^/m/z (abundance) | Fragment 2 MS2/ m/z (abundance) |
| --- | --- | --- | --- |
| 3-vinyl-[4H]-1,2-dithiin | 144 (49) | 111 (75) | 72 (25) |
| 2-vinyl-[4H]-1,3-dithiin | 144 (35) | 111 (34) | 72 (100) |
| (E), (Z)-Ajoene | 234(10) | 103 (91) | 45(100) |
